# Investigating the Effect of Nonrigid Connectors on the Success of Tooth-and-Implant-Supported Fixed Partial Prostheses in Maxillary Anterior Region: A Finite Element Analysis (FEA)

**DOI:** 10.1155/2021/5977994

**Published:** 2021-11-12

**Authors:** Ramin Mosharraf, Paria Molaei, Amirhossein Fathi, Sabire Isler

**Affiliations:** ^1^Department of Prosthodontics, Dental Materials Research Center, Dental Research Institute, School of Dentistry, Isfahan University of Medical Sciences, Isfahan, Iran; ^2^Dental Students' Research Committee, School of Dentistry, Isfahan University of Medical Sciences, Isfahan, Iran; ^3^Department of Prosthodontics, Dental Materials Research Center, Dental Research Institute, School of Dentistry, Isfahan University of Medical Sciences, Isfahan, Iran; ^4^Department of Prosthodontics, Faculty of Dentistry, Istanbul University, Istanbul, Turkey

## Abstract

**Objective:**

This study was designed to assess the effect of nonrigid connectors (NRCs) and their location in the success of tooth-and-implant-supported fixed prostheses in the maxillary anterior region by finite element analysis (FEA).

**Materials and Methods:**

Three 3D FEA models were designed, presuming maxillary lateral incisor and canine to be extracted. Implant (replacing canine), abutment, bone (spongious and cortical), central incisor (containing dentin, root cement, gutta-percha, and casting post and core), periodontal ligament, and three three-unit cemented PFM prostheses (a rigid one and two nonrigid) were modeled. The NRC was once in the tooth side and once in the implant side. The prostheses were loaded twice: 250N to the incisal edges (0° to the long axis) and 200 N to the cingula (45° to the long axis). The von-Mises stress and vertical displacement were analyzed.

**Results:**

Under both vertical and oblique loadings, the rigid model presented the highest stress. Under vertical loading, the NRC caused a significant decrease in the applied stress to the prosthesis, bone, implant, and tooth. Locating the NRC in the tooth side decreased the applied stress to the prosthesis and NRC. Under oblique loading, prosthesis and implant tolerated less stress in the presence of an NRC. Placing the NRC in the tooth side resulted in the least stress in all of the components except for porcelain and patrix. Vertical displacement of the tooth apex was approximately equal in all models.

**Conclusion:**

Using an NRC on the tooth side is the most efficient method in reducing the applied stress to prosthesis, implant, tooth, and bone. The amount of intrusion is not dependent on using an NRC or not.

## 1. Introduction

Implants have played an essential role in the edentulous patients' treatment since they were introduced by professor Brånemark [1]. In recent years, as the number of residual teeth has increased in the group of younger elderly, there has been an increasing interest in using fixed partial prostheses because of the higher quality of life they provide in comparison to removable dentures [2]. Both implant-supported and tooth-and-implant-supported fixed prostheses can be used for partially edentulous patients' oral rehabilitation. Due to the anatomic (e.g., insufficient available bone) or economic issues or the loss of osseointegration, inserting a second implant may be impossible, which leads to the lack of enough abutments to support an implant-supported fixed prosthesis [3–6]. So, a tooth-and-implant-supported fixed prosthesis may be considered as a treatment plan option. This kind of prosthesis may also be used to supply more support and stability for the prosthesis, to reduce the stress concentration around the implant, or to support distraction osteogenesis devices [6–8]. The periodontally compromised teeth which need more ferrulization or posterior edentulism, in which cantilevers are not allowed, are the other tooth-implant connection applications [3, 9]. It can also provide more bite force in comparison to the natural teeth [2].

The tooth-implant connection has always been a matter of controversy, and to date, there has been little agreement on whether tooth-and-implant can be connected and, if so, with which method [10, 11]. The main reason for this debate is the difference between the mobility of the natural teeth, which can move 50–200 *µ*m due to the presence of the periodontal ligament around it, and the ankylosed implant, which can only move 10 *µ*m as a result of bone flexibility [5, 8, 12]. Besides, the tooth and the implant have different movement patterns. The tooth has a two-phase movement, including a rapid movement and a linear one, while the implant has only a linear movement [13]. As a result, the prosthesis is considered to play the role of a cantilever and apply more force to the implant, which may lead to the stress convergence and bone loss around the implant neck, screw loosening and screw fracture in screw-retained implants, decementation of the prosthesis, or the abutment in cement-retained implants and implant fracture [9–16].

Although some practitioners consider tooth extraction as an alternative option to tooth-implant connection, Lindh rejected the idea of tooth extraction [8, 17]. On the other hand, using nonrigid connectors (NRC) or stress-absorbing elements was suggested to balance the mobility difference between the natural tooth and the implant [5, 10, 12, 18, 19]. However, the biggest concern about using nonrigid connectors is the intrusion phenomenon which may be seen [5, 6, 9, 13, 15, 17–20].

Some studies supported the idea of using rigid prostheses to avoid the intrusion of natural teeth as a result of using NRCs (e.g., Al-Omiri et al. [8], Gross and Laufer [10], Carillo et al. [17], Chee and Jivraj [21], and Ting et al. [22]), while some others argued that there is no significant difference between the tooth intrusion in the rigid and nonrigid prostheses (Breeding et al. [13], Garcia and Oesterle [23], and Ormianer et al. [24]).

Debate continues about the best strategies to connect the tooth and the implant in a fixed partial prosthesis. Besides, no research has been found that surveyed this subject in the anterior region. This study aimed to clarify several aspects of the tooth-and-implant-supported fixed partial prostheses in the maxillary anterior region. The research data in this study are drawn from three-dimensional finite element analysis (FEA). FEA is a method used for biomechanical analyses in the recent twenty years and makes us capable of analyzing stress and strain distribution in our assumed situation [5, 25]. In this study, von-Mises stress and vertical displacement were evaluated around different parts of prostheses with and without NRCs. Besides, the effects of the NRC location were also assessed. The null hypothesis was that nonrigid connectors were not effective in reducing the applied stress to the prosthesis and implant.

## 2. Materials and Methods

**Figure 1:**
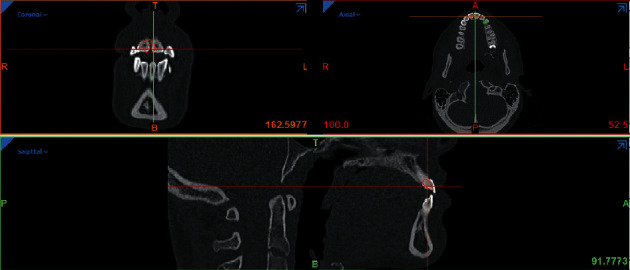
CT scan.

In order to assess the effect of nonrigid connectors (NRCs) and their location in the success of tooth-and-implant-supported fixed prostheses in the maxillary anterior region, three three-dimensional (3D) finite element models were designed for a partially edentulous maxilla. In this model, maxillary lateral incisor and canine were assumed to be extracted. The 3D models of the bone (both cortical and spongiose), periodontal ligament, enamel, dentin, and root cement were designed, using computed tomography (CT) (Figure 1), which were imported into mimics software with a 1 mm space between the slices and were improved manually by Mimics and 3matics softwares. Anterior maxillary ridge height was assumed to be 23 mm, cortical bone thickness as 1.5 mm, and the periodontal membrane was considered to be 0.3 mm thick. Casting post and core and three 3-unit PFM bridges were also designed in Mimics.

**Figure 2:**
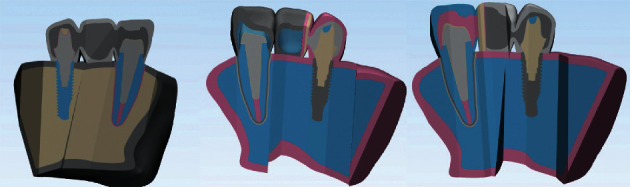
3D FEA models.

The dental implant and the abutment were designed in Solidworks 2020. A Straumann bone level tapered (BLT) implant with a diameter of 4.1 mm and a height of 10 mm and a cement-retained Straumann CARES titanium abutment with a gingival height of 1.5 mm were designed and placed in the maxillary canine location. The implant was assumed to be 100% osseo-integrated. The maxillary central was considered to be endodontically treated, one-third of the canal was filled with gutta-percha, and a casting post and core was designed for the tooth with 2 mm of the ferrule, which was cemented by zinc phosphate cement. The tooth was conventionally prepared for a PFM restoration. Three different 3-unit PFM bridges were modeled for the designed maxillary region. They were made of Ni–Cr alloy and porcelain and were cemented to the tooth-and-implant by zinc phosphate cement. The first model was a rigid restoration, and the next two models had slide-type NRCs. The NRC was once in the mesial side of the pontic, and once in its distal side, but in both models, the matrix part of the NRC was contained in the pontic (Figure 2). A friction coefficient with a value of 0.5 was considered between the matrix and the patrix. The models were transformed into Geomagic software before importing in Ansys software. The upper side of the maxilla was fixed. Once, 250 N static load was vertically applied to the incisal edge of the tooth, the implant crown, and the pontic. The next time, a 200 N static load was obliquely applied to the cingulum of the tooth, implant crown, and pontic (45° to the long axis). There were 10074 tetrahedral elements and 182373 nodes in the models. The elasticity modulus and Poisson's ratio of the pieces were defined according to the literature (Table 1) [5]. The components were considered to be homogenous, isotropic, and linear.

The models were analyzed by Ansys 2020 software, and the applied stress to the components and the vertical displacement of the natural teeth were measured and converted into color graphics.

## 3. Results

**Figure 3:**
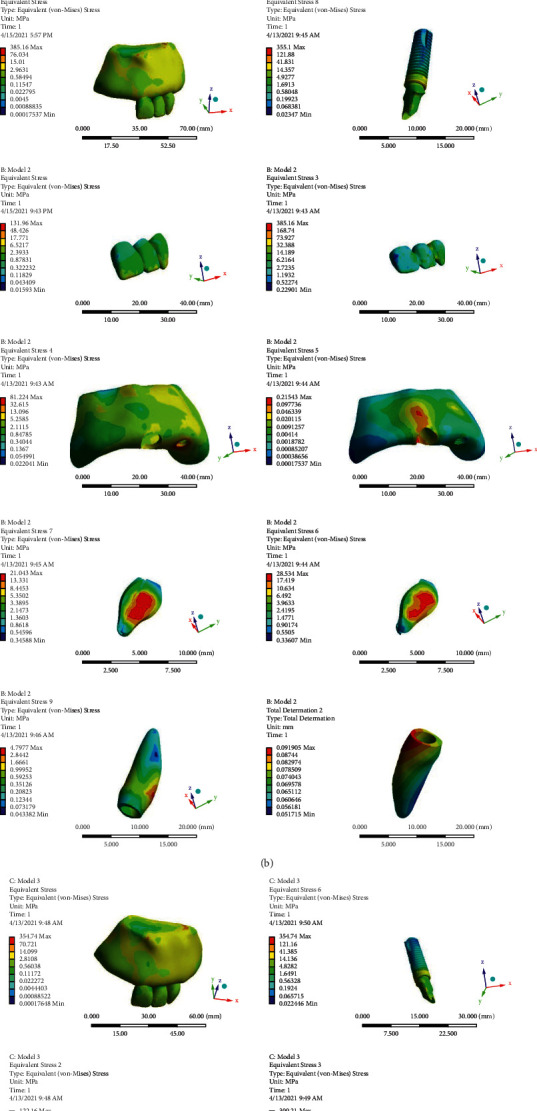
(a) Color spectrums manifest stress distribution of the entire model, implant, porcelain, metal frame, cortical bone, spongious bone and natural tooth (MPa) and the tooth vertical displacement (mm) under vertical loading in the first model. (b) Color spectrums manifest stress distribution of the entire model, implant, porcelain, metal frame, cortical bone, spongious bone, matrix, patrix and natural tooth (MPa) and the tooth vertical displacement (mm) under vertical loading in the second model. (c) Color spectrums manifest stress distribution of the entire model, implant, porcelain, metal frame, cortical bone, spongious bone, matrix, patrix and natural tooth (MPa) and the tooth vertical displacement (mm) under vertical loading in the third model.

**Figure 4:**
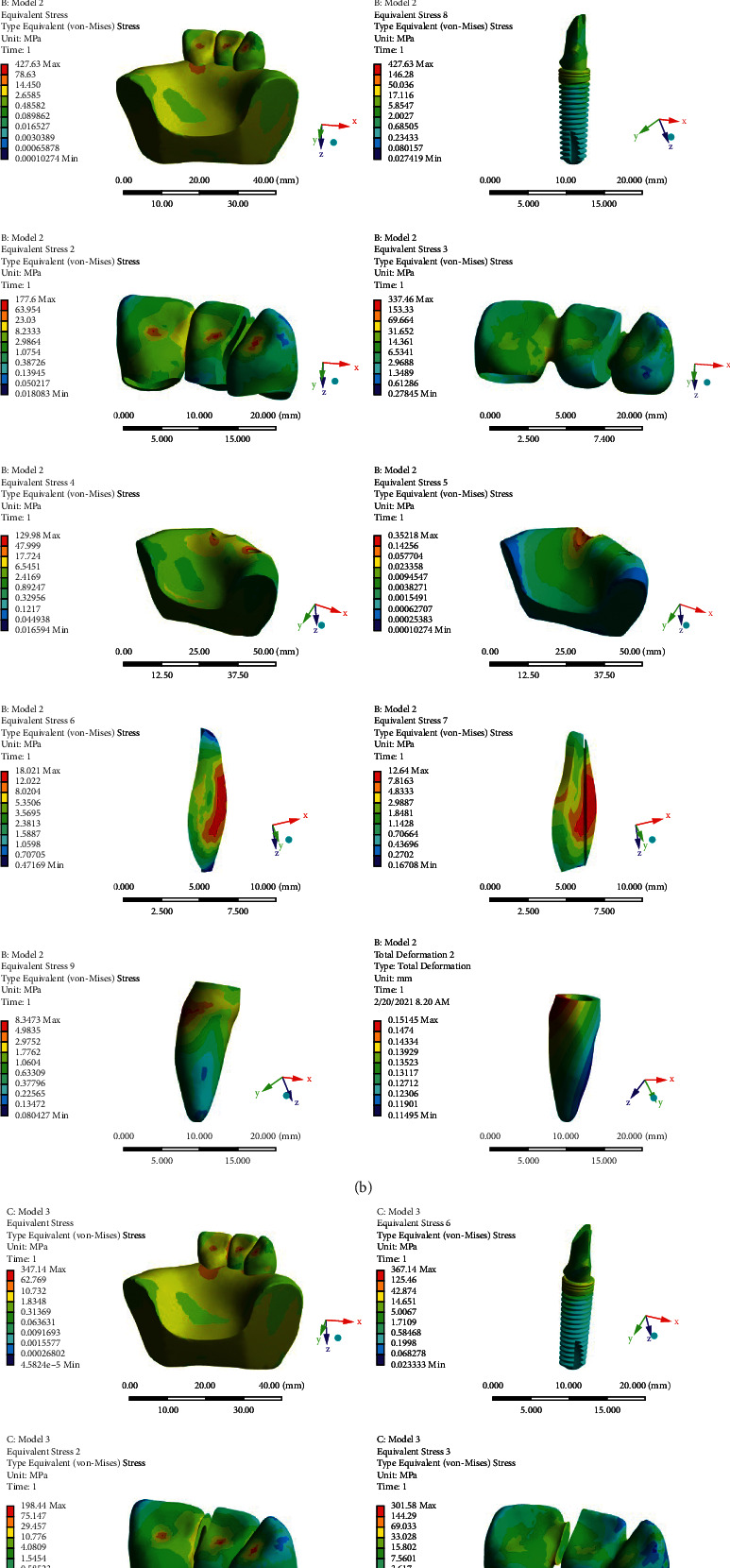
(a) Color spectrums depict stress distribution of the entire model, implant, porcelain, metal frame, cortical bone, spongious bone and natural tooth (MPa) and the tooth vertical displacement (mm) under oblique loading in the first model. (b) Color spectrums depict stress distribution of the entire model, implant, porcelain, metal frame, cortical bone, spongious bone, patrix, matrix and natural tooth (MPa) and the tooth vertical displacement (mm) under oblique loading in the second model. (c) Color spectrums manifest stress distribution of the entire model, implant, porcelain, metal frame, cortical bone, spongious bone, matrix, patrix and natural tooth (MPa) and the tooth vertical displacement (mm) under oblique loading in the third model.

- Model 1: a 3-unit PFM bridge in which the implant is rigidly connected to the natural tooth. The applied stress to the different parts of the model under vertical and oblique loadings are shown in Figures 3(a) and 4(a).
- Model 2: a 3-unit PFM bridge in which an NRC is located on the implant side. The applied stress to the different parts of the model under vertical and oblique loadings is shown in Figures 3(b) and 4(b).
- Model 3: a 3-unit PFM bridge in which an NRC is located on the tooth side. The applied stress to the different parts of the model under vertical and oblique loadings is shown in Figures 3(c) and 4(c).

The von-Mises stress and vertical displacement distribution of the models under vertical and oblique loadings are shown in Figures 3 and 4, subsequently.

The maximum applied von-Mises stresses to different components of the models are also given in Table 2.

The natural tooth vertical displacement in all of the models are shown in Figures 3 and 4, and maximum values of it in the apical area under both vertical and oblique loadings are given in Table 3.

## 4. Discussion

The present biomechanical study sets out with the aim of assessing the importance of using NRCs and their location in the success of the tooth-and-implant-supported fixed partial prostheses. Since previous studies have shown contradictory results and none of them have investigated this issue in the anterior region, this study evaluated the issue in this area.

As given in Table 2, under vertical load to the crowns' incisal edges, less stress was applied to the prosthesis (metal frame and porcelain), bone (cortical and spongious), implant, and natural tooth in the nonrigid models. The applied stress to the implant, natural tooth, and bone (cortical and spongious) were approximately equal in both of the models containing an NRC. Still, when the NRC was located near the natural tooth, less stress was applied to the prosthesis (metal frame and porcelain) and the NRC (matrix and patrix).

Additionally, under oblique loading to the crown's cingula, presence of the NRC caused a remarkable decrease in the applied stress to the prosthesis (metal frame and porcelain) and implant (Table 2). The von-Mises stress values of the spongious bone and the natural tooth in the rigid model were almost equal to the model with an NRC on the implant side and slightly more than the model in which the NRC was located in the tooth side. The maximum values of stress in the cortical bone were seen in the second model with an NRC attached to the implant, and the least one was seen in the third model with an NRC attached to the tooth. As the second and the third models were compared, all of the third model components tolerated less stress except for the porcelain and the patrix.

Applying stress to the natural tooth makes it intrude into the bone, as a result of periodontal ligaments presence, but the rigid ankylosed implant does not show this vertical displacement [5]. This difference in the movement between the tooth and implant may cause bending stress in the rigid prostheses. The remarkable decrease in the applied stress to the prosthesis, implant, and tooth is under both vertical and oblique loadings in the nonrigid models, and the concentration of stress in the NRC confirms the stress-absorbing character of the slide-type NRC which compensates the mobility difference between the implant and natural tooth. This result differs from previous studies that showed a remarkable increase in the prosthesis stress, when an NRC was used [19, 26]. The results also revealed that placing the NRC in the mesial side of the pontic is more efficient in decreasing the applied stress to the components under oblique loading. These findings do not support the previous research studies, which demonstrated that in the models, in which the NRC was placed on the implant side, the least amount of stress was observed [5, 12]. A possible explanation for this result might be that the NRC makes the prosthesis role as two different parts: a single crown on the central incisor and a cantilever on the implant (canine). According to the position of the central incisor in the maxillary arch, under oblique loading to the cingula, it has more anterior displacement in comparison to the canine. As a result, more stress may be seen in the prosthesis attached to the central incisor.

As given in Table 3, under vertical loading, the maximum vertical displacement of the apical area is almost equal in both of the nonrigid models and slightly less than the rigid one. Under oblique loading, maximum amount of vertical displacement of the apical area is nearly equal in all of the models. This result is consistent with those of Breeding et al. [13], Garcia and Oesterle [23], and Ormianer et al. [24] who declared that the intrusion is seen regardless of using NRCs or not.

The applied stress to the natural tooth is significantly less than the applied stress to the implant in all of the models, which confirms the stress-absorbing character of the periodontal ligament. On the other side, the stress distribution around the natural tooth-and-implant decrease from coronal to apical in all of the models, and the most amount of stress is applied to the cervical region of the tooth and the implant neck (Figures 3 and 4). This might be because of the loading location. As we take distance from the loading location, the stress values decrease. Another possible explanation for the stress concentration in the cervical area may be the difference in the elasticity modulus of the cortical and spongious bones [5].

Tooth-and-implant-supported fixed prostheses are difficult to evaluate because of their different and complicated elements, and their success rate is dependent on their biomechanical aspects [5]. These biomechanical aspects were analyzed by finite element analysis in this study. FEA can predict stress distribution around the different parts of the prosthesis under applied forces [25]. These data must be interpreted with caution because in vivo studies may show different results because of the complicated conditions in the oral environment. Although the ideal situation is when only vertical forces are applied to the tooth-and-implant long axis, the mastication forces are not only in one direction, neither are static [5]. Simulation of all of the applied forces to the prosthesis in the oral environment is not possible. Biomechanical features of the prosthesis under a vertical force to the tooth-and-implant long axis and an oblique one were analyzed in this study. The numerical values of this FEA study do not have a mathematical value and may differ in other models and under different forces.

Future studies on the current topic are therefore recommended. In future investigations, it may be possible to compare cantilevers with the rigid and nonrigid prostheses, using FEA. More in vivo studies on the subject are also recommended.

## 5. Conclusion

The present study was designed to determine the effect of using NRCs and their location in the success of tooth-and-implant-supported fixed prostheses treatment plan in the maxillary anterior region. Within the limitations of the study, the following conclusions can be drawn from it:

1. Using NRCs in the tooth-and-implant-supported fixed prostheses in the maxillary anterior region can significantly decrease the applied stress to the prosthesis, implant, natural tooth, and the bone
2. Putting the NRC on the tooth side is more efficient than placing it on the implant side in reducing stress values in the tooth-and-implant-supported fixed prostheses in the maxillary anterior region
3. Maximum values of the natural tooth movement and intrusion in the apical area are approximately equal in the prostheses with and without NRCs. The NRC location is not determinant either.

These findings provide further support for the hypothesis that prostheses with NRCs may be better choices compared to the prostheses in which the implant and the tooth are rigidly connected [27].

## Figures and Tables

**Table 1:** Materials' elasticity modulus (*E*) and Poisson's ratio (*v*).

Material properties	Elasticity modulus (*E*) (GPa)	Poison proportion (v)
Dentin	18.6	0.31
Implant	110	0.33
Cortical bone	15	0.30
Ni–Cr alloy	218	0.33
Enamel	84	0.33
Periodontal membrane	2	0.45
Porcelain	69	0.28
Spongiose bone	1.5	0.30
Nonrigid attachment	110	0.33
Zinc phosphate cement	22.4	0.35

**Table 2:** Maximum values of von-Mises stress (MPa) in the models with vertical incisal loading and oblique loading to the cingula.

Vertical loading								
	Porcelain	Metal frame	Cortical bone	Spongious bone	Implant	Natural tooth	Nonrigid connector matrix	Nonrigid connector patrix
Model 1	182.93	454.02	85.967	0.27093	444.85	5.9558	—	—
Model 2	131.96	385.16	81.224	0.21543	355.1	4.7977	21.043	28.534
Model 3	122.16	300.21	81.09	0.21532	354.74	4.7865	6.5591	7.5934

Oblique loading								
Model 1	267.94	664.97	109.51	0.35334	674.97	8.3127	—	—
Model 2	177.6	337.46	129.98	0.35218	427.63	8.3473	12.64	18.021
Model 3	198.44	301.58	75.477	0.32619	367.14	7.8435	11.364	26.533

**Table 3:** Maximum values of natural tooth vertical displacement in the apical area (mm).

	Under vertical loading	Under oblique loading
Model 1	0.08098	0.12422
Model 2	0.065112	0.12712
Model 3	0.065902	0.1234

## Data Availability

The data used to support the findings of this study are available from corresponding author upon request.
